# Single-cell RNA sequencing and integrated bioinformatics reveal new mitochondrial biomarkers in sarcopenia

**DOI:** 10.3389/fmolb.2026.1694362

**Published:** 2026-02-09

**Authors:** Hongan Ying, Wenhan Wang, Lili Huang, Weiwen Hong, Lingchang Yang

**Affiliations:** 1 Department of Geriatrics, Traditional Chinese Medicine Hospital of Huangyan, Taizhou, China; 2 Department of General Surgery, Traditional Chinese Medicine Hospital of Huangyan, Taizhou, China; 3 Department of Emergency, Taizhou First People’s Hospital, Taizhou, China; 4 Department of Emergency, Traditional Chinese Medicine Hospital of Huangyan, Taizhou, China

**Keywords:** biomarkers, diagnosis, mitochondrial dysfunction, sarcopenia, single-cell RNA sequencing

## Abstract

**Background:**

Sarcopenia, characterized by age-related skeletal muscle loss and dysfunction, affects approximately 10% of adults over 60 years worldwide. Current diagnostic methods often detect sarcopenia only after substantial muscle deterioration has occurred, highlighting the critical need for early diagnostic biomarkers.

**Methods:**

We conducted an integrated analysis of several public transcriptomic datasets (GSE1428, GSE117525, GSE167186, GSE111006, GSE111010, and GSE111016) employing differential gene expression analysis, weighted gene co-expression network analysis, and machine learning techniques. Single-cell RNA sequencing (scRNA-seq) was employed to determine cell type-specific expression. Quantitative PCR validated the findings in C2C12 myoblasts cultured under sarcopenia-like conditions. A nomogram-based predictive model was developed and assessed through ROC analysis and decision curve analysis.

**Results:**

We discovered a conserved three-gene mitochondrial signature (CHCHD10, SAMM50, MDH2) significantly dysregulated across multiple independent cohorts. Single-cell RNA sequencing identified distinct expression patterns across cell types, highlighting significant mitochondrial changes in myocytes. A nomogram model integrating these three genes demonstrated superior diagnostic accuracy (AUC = 0.883, 95% CI: 0.732–1.000) compared to conventional clinical parameters. *In vitro* validation confirmed significant downregulation of all three biomarkers in a sarcopenia-like state (CHCHD10, p < 0.01; SAMM50, p < 0.05; MDH2, p < 0.01).

**Conclusion:**

Our findings suggest that a three-gene mitochondrial signature, comprising CHCHD10, SAMM50, and MDH2, could serve as a valuable biomarker for early sarcopenia diagnosis. This signature underscoring the contribution of mitochondrial dysfunction to muscle aging. By potentially bridging basic research with clinical application, this panel may offer novel targets for developing mitochondria-targeted therapies and monitoring their efficacy.

## Introduction

Sarcopenia, marked by the age-related decline in skeletal muscle mass and function, is a significant and increasing global health issue impacting about 10% of adults over 60 years old worldwide (Shafiee et al., 2017). Sarcopenia, as defined by the European Working Group on Sarcopenia in Older People (EWGSOP2), is a progressive disorder characterized by diminished muscle strength, decreased muscle mass or quality, and compromised physical performance. Sarcopenia imposes a significant economic burden, with annual healthcare costs estimated at $18. Five billion in the United States alone (Janssen et al., 2004). Sarcopenia significantly impacts patients’ quality of life, elevating the risk of falls by 2.5 times, fractures by 3 times, and contributing to functional decline, hospitalization, and early mortality (Landi et al., 2012; Beaudart et al., 2017). The condition’s pathophysiology is multifactorial, involving neuromuscular junction degeneration, protein synthesis/degradation imbalance, hormonal changes, and chronic inflammation (Rodrigues et al., 2022). As global populations age, sarcopenia’s prevalence is projected to increase dramatically, necessitating improved diagnostic and therapeutic approaches (Ethgen et al., 2017).

Sarcopenia diagnosis predominantly involves assessing muscle mass through dual-energy X-ray absorptiometry (DXA) or bioelectrical impedance analysis (BIA), evaluating muscle strength with handgrip dynamometry, and measuring physical performance using gait speed or chair rise tests (Cruz-Jentoft et al., 2019). A critical limitation of these methods is their inability to detect sarcopenia in its early, preclinical stages; they typically identify the condition only after substantial and often irreversible muscle loss has occurred (Calvani et al., 2015). The search for reliable biomarkers has yielded several candidates, including inflammatory markers (IL-6, TNF-α, CRP), hormonal factors (testosterone, IGF-1), and myokines (myostatin, irisin) (Chen et al., 2014; Liu et al., 2024). However, these candidates largely lack the requisite sensitivity and specificity for reliable early diagnosis or have proven difficult to translate into routine clinical practice (Chen et al., 2014). Recent advances in-“omics” technologies have identified potential molecular signatures of sarcopenia, but translation into clinical applications remains limited (Sanchez-Rodriguez et al., 2020). Furthermore, existing diagnostic criteria exhibit poor consistency across ethnic and clinical populations, underscoring the urgent need for universally applicable, objective biomarkers (Cruz-Jentoft et al., 2014). Despite extensive research, the early molecular events preceding clinical sarcopenia remain poorly understood (Marzetti et al., 2017).

Among the various pathophysiological mechanisms, mitochondrial dysfunction has emerged as a central player in the pathogenesis of sarcopenia (Marzetti et al., 2013). Aging muscle demonstrates marked reductions in mitochondrial content, with up to 40% decrease in mitochondrial DNA and oxidative capacity by the eighth decade of life (Short et al., 2005). Several key mitochondrial alterations contribute to sarcopenia: impaired biogenesis regulated by PGC-1α, compromised quality control through disrupted mitophagy, increased oxidative damage from reactive oxygen species (ROS), and dysregulated apoptotic signaling (Joseph et al., 2012; Calvani et al., 2013). Studies have shown that age-related declines in complex I and IV activities correlate with muscle mass loss and functional impairment (Bua et al., 2006). Mitochondria also integrate multiple cellular stress signals relevant to sarcopenia, including inflammation, nutrient availability, and mechanical stimuli (Romanello and Sandri, 2016). This progressive mitochondrial dysfunction culminates in a diminished capacity for adaptation, thereby blunting the efficacy of therapeutic interventions such as exercise and nutritional supplementation in older adults (Hood et al., 2019). Recent evidence suggests that specific mitochondrial pathways may differ in their contribution to fiber type-specific muscle loss, a limitation largely attributable to the reliance of most previous studies on bulk tissue analysis, which masks critical cell-type-specific contributions and heterogeneity (Katic et al., 2007). Despite compelling evidence linking mitochondrial dysfunction to sarcopenia, a systematic approach to identify and validate mitochondrial-derived biomarkers for diagnostic or prognostic applications is lacking (Gonzalez-Freire et al., 2015). This study utilizes integrated bioinformatics and single-cell RNA sequencing (scRNA-seq) to establish a precise mitochondrial gene signature for sarcopenia, overcoming the limitations of bulk tissue analysis. This method seeks to offer new insights into the cellular mechanisms of muscle wasting and produce biomarkers for early detection.

## Methods

### Data sources and preprocessing

This research employed publicly accessible gene expression datasets from the Gene Expression Omnibus (GEO) database (https://www.ncbi.nlm.nih.gov/geo/) to examine mitochondrial gene signatures associated with sarcopenia. The dataset selection process began with a comprehensive search using keywords “sarcopenia,” “skeletal muscle,” and “aging,” followed by application of specific inclusion criteria: confirmed sarcopenia diagnosis based on established clinical guidelines, age-matched control samples, adequate sample size, and comprehensive gene expression coverage on the respective platforms.

After rigorous quality assessment evaluating data completeness, normalization consistency, and platform compatibility, six datasets (GSE1428, GSE117525, GSE167186, GSE111006, GSE111010, and GSE111016) were selected for downstream analysis (Table 1). These datasets collectively comprised 100 sarcopenia samples and 189 control samples across different microarray platforms. The GSE1428 dataset (GPL96 platform) included 12 sarcopenia and 10 control samples, while the largest dataset, GSE167186 (GPL2467), contained 24 sarcopenia and 52 control samples.

**TABLE 1:** Provides a concise overview of microarray data.

GEO number	Platform	Disease samples	Control samples	Organism
GSE1428	GPL96	12	10	*Homo sapiens*
GSE117525	GPL20880	31	41	*Homo sapiens*
GSE167186	GPL2467	24	52	*Homo sapiens*
GSE111006	GPL16791	4	36	*Homo sapiens*
GSE111010	GPL16791	9	30	*Homo sapiens*
GSE111016	GPL16791	20	20	*Homo sapiens*
GSE269698	GPL24247	6	6	*Mus musculus*

We utilized the ComBat algorithm from the sva R package (version 3.42.0) to address batch effects and platform-specific variations. The raw expression data underwent log2 transformation and, if required, quantile normalization. Probe annotations were updated to current gene symbols using the annotate and org.Hs.e.g.,.db R packages. For genes with multiple probes, the probe exhibiting the highest average expression was selected. Our study concentrated on genes associated with mitochondria, sourced from the MitoCarta 3.0 database (https://www.broadinstitute.org/mitocarta/mitocarta30- inventory- mammalian- mitochondrial-proteins-and-pathways). Results were visualized using the ggplot2 (v3.3.6) and pheatmap (v1.0.12) packages in R.

### Screening of key genes

**FIGURE 1:**
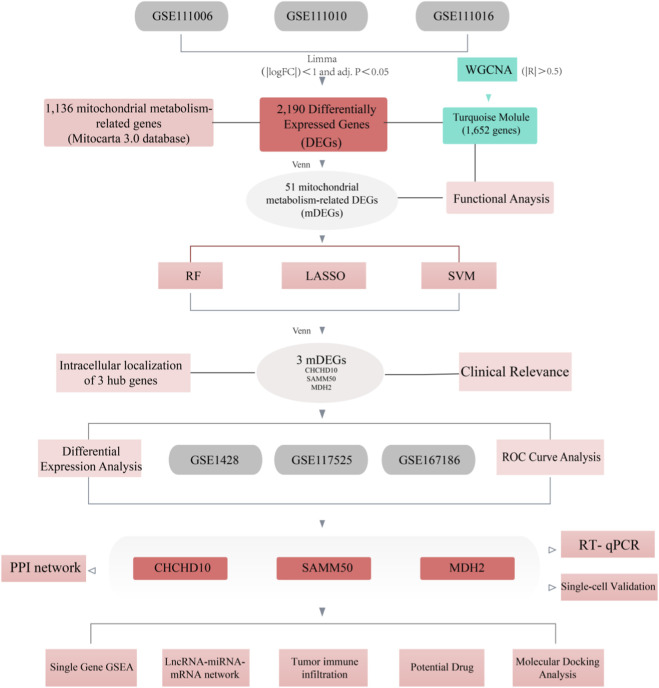
Illustrates the analysis process flowchart.

To identify robust diagnostic biomarkers for sarcopenia, we employed a multi-step screening strategy. First, Differentially Expressed Genes (DEGs) between the sarcopenia and control groups were identified using the 'limma’ package (criteria: |log2FC| > 1 and adjusted P-value <0.05). These DEGs were then intersected with module genes identified via Weighted Gene Co-expression Network Analysis (WGCNA) and mitochondrial-related genes from the MitoCarta 3.0 database. Subsequently, three machine learning algorithms—Least Absolute Shrinkage and Selection Operator (LASSO) regression, Random Forest (RF), and Support Vector Machine-Recursive Feature Elimination (SVM-RFE)—were applied to the intersecting genes to reduce dimensionality and pinpoint candidate features.4 Finally, the diagnostic efficacy of the selected hub genes was evaluated using Receiver Operating Characteristic (ROC) curve analysis. Genes with an Area Under the Curve (AUC) greater than 0.7 were retained as the final key diagnostic biomarkers, adhering to the standard for acceptable diagnostic accuracy (Figure 1).

### Weighted gene co-expression network analysis (WGCNA)

WGCNA is a method used to identify clusters (modules) of highly correlated genes, summarize these clusters using the module eigengene or an intramodular hub gene, and relate modules to one another and to external sample traits. This approach facilitates the identification of candidate biomarkers or therapeutic targets by focusing on the interconnectedness of gene expression profiles.

The WGCNA R package (v1.70–3) was used to identify gene co-expression modules. A signed network was constructed using an appropriate soft thresholding power (β) to achieve scale-free topology (*R* ^2^ > 0.55) after filtering out low-variance genes. The adjacency matrix was converted into a topological overlap matrix (TOM), and modules were detected using hierarchical clustering with dynamic tree cutting, setting a minimum module size of 30 genes and a merging threshold of 0.25.

Module-trait relationships were evaluated by correlating module eigengenes with clinical traits. Modules with p-values less than 0.05 were chosen for additional analysis. Hub genes were determined using criteria of gene significance (GS) greater than 0.2 and module membership (MM) exceeding 0.8. Key modules underwent functional enrichment analysis using GO and KEGG pathways via the clusterProfiler package, with significance set at adjusted p < 0.05.

### Analysis of protein-protein interaction (PPI) networks

We utilized the STRING database (v11.5) to construct protein-protein interaction networks, aiming to analyze the functional interactions among the mitochondrial biomarkers CHCHD10, SAMM50, and MDH2. The three biomarkers served as seed nodes, and we included first-order interacting partners with a combined confidence score ≥0.7. Only experimentally validated and database-curated interactions were retained for analysis.

Network topology was analyzed using Cytoscape (v3.9.1) with the NetworkAnalyzer plugin. We calculated degree, betweenness, and closeness centrality measures to identify key proteins within the network. Functional modules were detected using the MCODE algorithm and subsequently annotated using Gene Ontology enrichment analysis.

Proteins were categorized as mitochondrial, mitochondrial-associated, or non-mitochondrial using the MitoCarta 3.0 database. The final network was visualized with node sizes representing connectivity degree and the three biomarker proteins highlighted to emphasize their position within the interaction landscape.

### Single-sample gene set enrichment analysis (ssGSEA)

We employed ssGSEA via the GSVA package (version 1.42.0) in R to analyze functional pathways affected by sarcopenia at the individual sample level. This approach evaluates pathway enrichment by ranking genes according to their expression values in each normalized expression matrix. Separate enrichment scores were computed for each sample-pathway pair, allowing for personalized assessment of pathway alterations.

We assessed enrichment across various functional databases, such as Gene Ontology (GO) terms (encompassing Biological Process, Molecular Function, and Cellular Component), Kyoto Encyclopedia of Genes and Genomes (KEGG) pathways, and Reactome pathways. The algorithm employed a modified Kolmogorov-Smirnov statistic to compute enrichment scores, representing the degree to which genes in a given pathway were coordinately up- or downregulated within each sample.

The resulting sample-specific enrichment scores were used to identify pathways with differential activity between experimental conditions and to correlate pathway activities with clinical parameters. Pathway enrichment scores between groups were compared using t-tests or Mann-Whitney U tests, as suitable. P-values were adjusted for multiple comparisons with the Benjamini-Hochberg method, considering FDR <0.05 as significant.

### Immune cell infiltration patterns

To characterize the immune microenvironment associated with sarcopenia, we performed immune cell infiltration analysis using transcriptomic data. Cell-type deconvolution utilized the CIBERSORT algorithm and the LM22 signature matrix to identify 22 immune cell subtypes. Gene expression profiles from sarcopenia muscle samples and healthy controls were analyzed using the CIBERSORT web portal (https://cibersort. stanford. edu) with 1,000 permutations.

For validation, we applied an independent method, xCell, which uses a gene signature-based approach to infer the abundance of 64 cell types from bulk RNA-seq data. The Mann-Whitney U test, adjusted with the Benjamini-Hochberg method for multiple comparisons, was employed to compare relative immune cell proportions between sarcopenia and control groups, with significance set at adjusted p < 0.05.

Spearman’s rank correlation was used to analyze the relationship between specific immune cell abundances and mitochondrial biomarkers (CHCHD10, SAMM50, and MDH2). Results were visualized as heatmaps and box plots using the ggplot2 package in R (v4.1.2).

### Competitive endogenous RNA (ceRNA) network construction

To construct a competitive endogenous RNA (ceRNA) network centered on our three mitochondrial biomarkers (CHCHD10, SAMM50, and MDH2), we identified potential regulatory miRNAs using three complementary databases: miRDB, TargetScan, and miRWalk. Only miRNA-mRNA interactions predicted by at least two databases with high confidence scores (miRDB target score ≥80, TargetScan context++ score ≤ −0.3) were retained. For upstream circRNA regulation, we utilized the circBase and starBase databases to identify circRNAs with validated miRNA binding sites matching our selected miRNAs.

The resulting miRNA-mRNA-circRNA interactions were integrated into a comprehensive regulatory network using Cytoscape (v3.9.1). Node sizes represented the degree of connectivity, with edge weights corresponding to prediction confidence scores. This multilevel network approach provided insights into the potential post-transcriptional regulatory mechanisms affecting our mitochondrial biomarkers in sarcopenia.

### Single-cell RNA sequencing analysis

Single-cell RNA sequencing data were sourced from the Human Muscle Ageing Cell Atlas (HMA) database (https://db. cngb. org/cdcp/hlma/). The raw sequencing data (accession numbers CNP0004394, CNP0004395, CNP0004494, CNP0004495) underwent filtering, demultiplexing, and alignment to the hg38 reference genome (Lai et al., 2024).

Cells were filtered using standard quality control metrics (UMIs >1,000, genes >500, mitochondrial content <5%). Doublets were eliminated using DoubletFinder (v. 2.0.3), and ambient RNA was minimized with SoupX (v.1.4.8). Data underwent normalization, log transformation, and Harmony was used to correct batch effects between snRNA-seq and scRNA-seq.

Unsupervised clustering was performed using the Louvain algorithm, and cell types were annotated based on canonical markers. Seurat (v.4.0.2) was employed for differential expression analysis across age groups, applying thresholds of log_2_ [fold change] > 0.25 and Q < 0.05. Functional enrichment was performed using Metascape.

Trajectory analysis was implemented with Monocle3 to study myofiber degeneration. Cell-cell interactions were analyzed using CellChat (v.1.1.0). Age-related changes in cell composition were modeled using a generalized linear mixed model, accounting for biological and technical covariates.

### Predictive model development and validation

A visual prediction tool was developed using the “rms” package in R, integrating the three mitochondrial biomarkers into a nomogram. Calibration curves and the Hosmer-Lemeshow test were used to evaluate the alignment between predicted probabilities and observed outcomes. Decision Curve Analysis (DCA) assessed the predictive model’s clinical utility by determining net benefit at different threshold probabilities. The model’s discriminative ability was evaluated through ROC curve analysis, with the AUC used to quantify its performance. Ten-fold cross-validation was employed to ensure robust estimation of model performance metrics. Statistical analyses were performed using R software (version 4.1.2), with significance set at p < 0.05.

### Molecular docking analysis

We utilized AutoDock Vina 1.2.2 for molecular docking analysis to discover therapeutic compounds targeting mitochondrial biomarkers CHCHD10, SAMM50, and MDH2. The crystal structures of the three biomarker proteins were obtained from the RCSB Protein Data Bank. A library of 1,500 FDA-approved small molecules was obtained from the Drug Signatures Database (DSigDB), with 3D structures collected from PubChem.

Protein structures were processed by eliminating water molecules, incorporating polar hydrogens, and calculating Gasteiger charges. Ligand files were prepared with rotatable bonds defined and energy minimized. A grid box of 25 × 25 × 25 Å centered on each protein’s predicted active site was used for docking simulations. For each protein-ligand pair, 10 independent docking runs were performed using default exhaustiveness settings. Binding poses were ranked by binding affinity (kcal/mol), with binding affinities < −7.0 kcal/mol typically indicating strong binding. The top-scoring compounds were further analyzed for hydrogen bonding patterns and hydrophobic interactions using PyMOL.

### PCR validation

The C2C12 mouse myoblasts cell line present in this study were obtained from Wuhan Pricella Biotechnology Co., Ltd. (catalog number: PC-H2024093015). C2C12 were maintained in DMEM supplemented with 10% fetal bovine serum (FBS) and 1% penicillin-streptomycin (P/S) at 37 °C in a humidified atmosphere containing 5% CO_2_. Cells were plated in 6-well plates at a density of 2 × 10^5^ cells per well and cultured overnight to reach about 90% confluence. The cells were then divided into control and differentiation groups. To construct the *in vitro* sarcopenia model, fully differentiated myotubes were treated with 5 μM Dimethylsulfoxide (DMSO) for 24 h, while the control group was treated with an equal volume of vehicle ethanol (Eo et al., 2020). Total RNA was isolated with Trizol reagent according to the manufacturer’s instructions. RNA purity and concentration were evaluated using a NanoDrop-2000 spectrophotometer, with acceptable A260/A280 ratios ranging from 1.8 to 2.0. RNA (1 µg) was reverse-transcribed with SuperRT III All-in-one RT Mix.

Quantitative PCR was performed on a LightCycler 480 (Roche) using 2× M5 HiPer SYBR Premix Es Taq. PCR cycling conditions included an initial step at 95 °C for 2 min, followed by 45 cycles of 95 °C for 5 s and 60 °C for 30–34 s. Triplicate analyses were performed for each sample. Primers for mitochondrial biomarkers included: CHCHD10 (F: TGGGGGAAATTCAGAGCCTG, R: CCTCACA CAGGGTTAGGTCG), SAMM50 (F: GCAGTATGGTACTCGGCCTC, R: CTCCTCTGTCTGTCTC CCGA), and MDH2 (F: TTCAACACCAACGCTACCATTGTG, R: GTGTTCGCTCTGACGATGTCA AGG). The reference gene utilized was β-actin. Relative expression was determined using the 2^(-ΔΔCT)^ method, and statistical analysis was conducted with GraphPad Prism.

### Statistical analysis

R software (version 4.2.1) was used for statistical analyses. Group differences were evaluated using Student’s t-test for normally distributed data and the Mann-Whitney U test for non-parametric data. One-way ANOVA with Tukey’s post-hoc test was used for multiple group comparisons. The Benjamini-Hochberg procedure was employed to maintain the false discovery rate (FDR) below 0.05 for statistical significance in multiple testing. Pearson’s or Spearman’s correlation coefficients were used for correlation analyses, depending on suitability. Kaplan-Meier curves and log-rank tests were utilized for survival analyses. Differential gene expression was analyzed using the limma package. Data are expressed as mean ± standard deviation (SD), with statistical significance set at p < 0.05. Graphs were generated using ggplot2 package in R.

## Results

### Analysis of sarcopenia-related gene expression datasets

**FIGURE 2:**
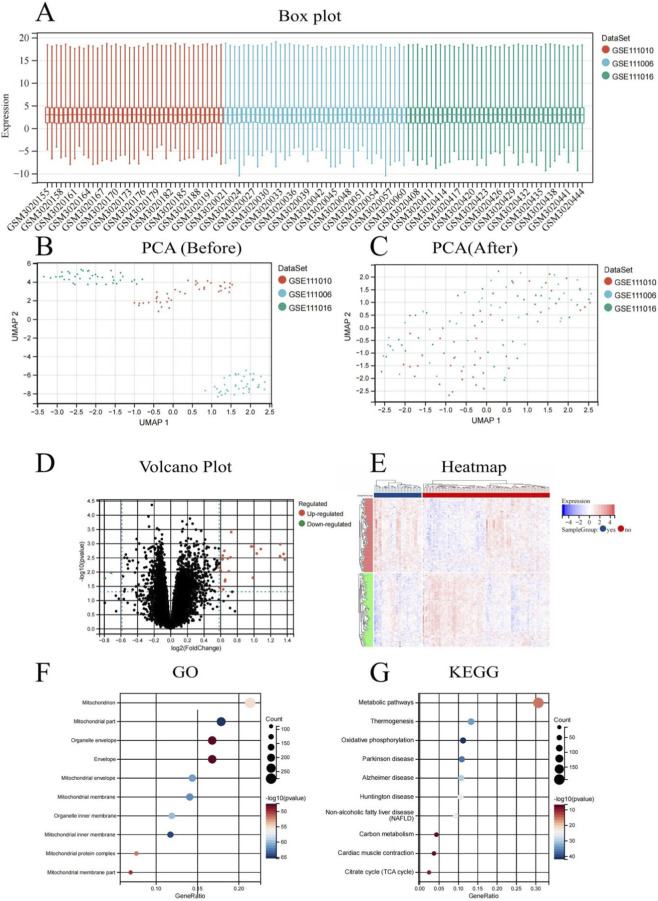
Integrated bioinformatics analysis of sarcopenia-related gene expression profiles from GEO datasets (GSE111006, GSE111010, and GSE111016). **(A)** Box plot illustrating the distribution of normalized gene expression values across all samples from the three datasets. **(B)** Principal component analysis (PCA) before batch effect correction, demonstrating high dispersion and clear separation between datasets. **(C)** PCA after batch effect correction, showing improved data integration and reduced dataset-specific clustering. **(D)** Volcano plot illustrating differentially expressed genes (DEGs) between sarcopenia and control groups, identified via the limma method. Red dots indicate significantly upregulated genes, blue dots denote significantly downregulated genes, and gray dots represent non-significant genes. **(E)** Heatmap depicting the expression profiles of DEGs across all samples, with columns representing individual samples and rows representing genes. Red signifies elevated expression levels, while blue denotes reduced expression levels. **(F)** Gene Ontology (GO) enrichment analysis of differentially expressed genes (DEGs) is depicted, where dot size indicates the number of genes and color intensity reflects the level of statistical significance. **(G)** KEGG pathway enrichment analysis of DEGs, highlighting dysregulated biological pathways in sarcopenia.

We conducted an integrated analysis of three GEO datasets (GSE111006, GSE111010, and GSE111016) to thoroughly explore the molecular mechanisms of sarcopenia. The normalization process successfully aligned gene expression distributions across samples, as indicated by uniform median values in the box plots (Figure 2A). Initial principal component analysis (PCA) without normalization indicated significant batch effects and considerable variability among datasets Figure 2B, whereas post-normalization PCA demonstrated markedly improved data integration with reduced batch effects, allowing for more reliable downstream analyses (Figure 2C).

Differential expression analysis using the limma method identified significant transcriptomic alterations between sarcopenia and control groups. The volcano plot (Figure 2D) illustrates the distribution of differentially expressed genes (DEGs), with numerous significantly upregulated (red) and downregulated (blue) genes. The expression patterns of these DEGs across all samples were visualized in a heatmap (Figure 2E) demonstrates distinct clustering of sarcopenia and control samples according to gene expression profiles.

Functional annotation of DEGs through Gene Ontology (GO) enrichment analysis highlighted significant enrichment in mitochondrial components and functions (Figure 2F). The top enriched GO terms included mitochondrion, mitochondrial part, organelle envelope, mitochondrial envelope, and mitochondrial membrane, suggesting profound alterations in mitochondrial biology in sarcopenia. KEGG pathway analysis (Figure 2G) further supported these findings, showing significant enrichment in metabolic pathways, thermogenesis, oxidative phosphorylation, and various neurodegenerative diseases such as Parkinson’s, Alzheimer’s, and Huntington’s. This intriguing overlap suggests shared molecular mechanisms between sarcopenia and neurodegenerative diseases, potentially centering on mitochondrial failure. The enrichment of pathways such as non-alcoholic fatty liver disease (NAFLD), carbon metabolism, cardiac muscle contraction, and the citrate cycle (TCA cycle) suggests extensive metabolic dysregulation in sarcopenia.

### Analysis of sarcopenia-related genes using WGCNA

**FIGURE 3:**
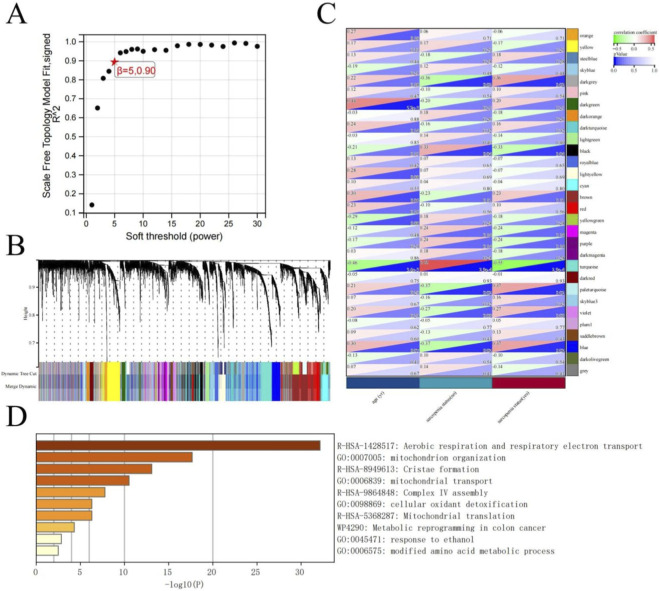
Analysis of sarcopenia-associated genes using Weighted Gene Co-expression Network Analysis. **(A)** Scale-free topology model fit for soft threshold power selection. The graph illustrates the correlation between the soft threshold (power) and the scale-free topology fitting index (*R* ^2^), indicating that the chosen threshold β = 5 results in an *R* ^2^ of 0.90. **(B)** Hierarchical clustering dendrogram of genes illustrating module identification via dynamic tree cutting and merging. Different colors represent distinct co-expression modules. **(C)** A heatmap illustrating the correlations between gene modules (rows) and clinical traits (columns). Each cell displays the correlation coefficient alongside its p-value. Red signifies a positive correlation, while blue denotes a negative one, with color intensity reflecting the correlation’s strength. The turquoise module shows the strongest association with sarcopenia status. **(D)** Functional enrichment analysis was conducted on 1,652 genes within the turquoise module. The bar chart shows the top enriched biological processes and pathways, with bar length representing statistical significance (-log10(P)). The analysis reveals significant enrichment in mitochondrial functions and metabolic processes.

We conducted WGCNA to clarify the functional relationships among genes associated with sarcopenia. A soft threshold power of β = 5 (*R* ^2^ = 0.90) was chosen for constructing a scale-free network, as determined by the scale-free topology model fit analysis. Figure 3A. This threshold optimally balanced the scale-free network properties while preserving strong gene connections.

Hierarchical clustering with dynamic tree cutting identified distinct gene co-expression modules, visualized as different colors in the dendrogram (Figure 3B). After merging, we identified 30 stable modules, each representing a group of genes with highly correlated expression patterns. The module-trait relationship analysis (Figure 3C) revealed several modules significantly associated with sarcopenia status. Notably, the turquoise module exhibited the strongest positive correlation with sarcopenia (correlation coefficient = 0.55, p-value = 2.9e-4), while showing a significant negative correlation with the non-sarcopenia status (correlation coefficient = −0.55, p-value = 2.9e-4).

Functional enrichment analysis of the 1,652 genes within the turquoise module (Figure 3D) revealed significant enrichment in mitochondrial processes and metabolic pathways. The most significantly enriched terms included aerobic respiration and respiratory electron transport (R-HSA-1428517), mitochondrion organization (GO:0007005), cristae formation (R-HSA-8949613), and mitochondrial transport (GO:0006839). Additional enriched pathways included Complex IV assembly (R-HSA-9864848), cellular oxidant detoxification (GO:0098869), and mitochondrial translation (R-HSA-5368287). Non-mitochondrial processes were also identified, including metabolic reprogramming in colon cancer (WP4290), response to ethanol (GO:0045471), and modified amino acid metabolic processes (GO:0006575). The functional profile of the turquoise module unequivocally establishes mitochondrial dysfunction, particularly in electron transport and organization, as a cornerstone of sarcopenia pathogenesis.

These findings underscore the pivotal role of mitochondrial dysfunction and metabolic alterations in sarcopenia pathogenesis, offering potential therapeutic targets.

### Identification and characterization of key mitochondrial genes in sarcopenia using machine learning approaches

**FIGURE 4:**
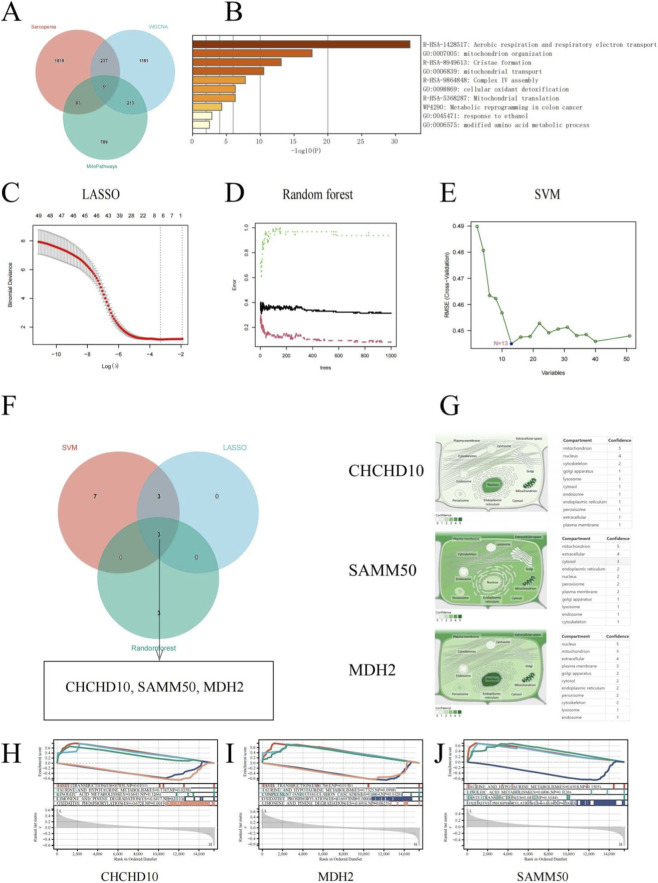
Identification and characterization of core mitochondrial genes associated with sarcopenia using machine learning approaches. **(A)** Venn diagram showing the overlap between differentially expressed genes from merged GEO datasets, genes in the turquoise module from WGCNA analysis, and mitochondrial genes from the MitoCarta3.0 database. The intersection identifies 51 genes common to all three sets. **(B)** Functional enrichment analysis of the 51 overlapping genes, showing significant enrichment in mitochondrial processes. **(C)** LASSO regression analysis with cross-validation for optimal lambda parameter selection, identifying 13 genes with non-zero coefficients (at λ with minimum cross-validation error). **(D)** Random forest error rate optimization, showing the relationship between the number of trees and error rates, with optimal performance at 30 trees. **(E)** Utilize Support Vector Machine (SVM) for feature selection by plotting the root mean square error (RMSE) against the count of variables incorporated in the model. **(F)** Venn diagram illustrates the gene overlap identified by three machine learning methods: LASSO, Random Forest, and SVM. The genes CHCHD10, SAMM50, and MDH2 were consistently selected by all three approaches. **(G)** Subcellular localization of CHCHD10, SAMM50, and MDH2 based on the GeneCards database, indicate their mitochondrial localization. **(H–J)** ssGSEA was conducted for CHCHD10, SAMM50, and MDH2. The upper portion of each panel shows the ranked gene list metric, while the lower portion displays the enrichment score plots for the top positively and negatively enriched pathways. Notably, oxidative phosphorylation is consistently negatively enriched across all three genes.

We employed an integrative multi-omics strategy, incorporating differential expression analysis, co-expression network analysis, and mitochondrial database mining, to pinpoint key mitochondrial genes associated with sarcopenia. The intersection of differentially expressed genes from our merged GEO datasets (3,019 genes), genes within the sarcopenia-associated turquoise module from WGCNA (1,652 genes), and known mitochondrial genes from the MitoCarta3.0 database (1,140 genes) yielded 51 high-confidence mitochondrial sarcopenia-associated genes (Figure 4A).

Functional enrichment analysis of these 51 genes (Figure 4B) confirmed their significant involvement in critical mitochondrial processes, particularly aerobic respiration and respiratory electron transport, mitochondrion organization, cristae formation, and mitochondrial transport. This analysis reinforced the pivotal role of mitochondrial dysfunction in the pathophysiology of sarcopenia.

To further narrow down the list to the most crucial genes, we employed three complementary machine learning approaches. LASSO regression with optimized lambda parameter selection identified 13 genes with non-zero coefficients that best discriminated between sarcopenia and control samples (Figure 4C). Random forest analysis determined optimal model performance at 30 trees with minimal error rates (Figure 4D), while Support Vector Machine (SVM) feature selection identified key discriminatory genes (Figure 4E). Remarkably, three genes—CHCHD10, SAMM50, and MDH2—were consistently selected by all three machine learning methods (Figure 4F), suggesting their central importance in sarcopenia.

Subcellular localization analysis based on the GeneCards database confirmed the mitochondrial localization of all three genes (Figure 4G). CHCHD10 is found in the mitochondrial intermembrane space, SAMM50 is an integral protein of the outer mitochondrial membrane, and MDH2 resides in the mitochondrial matrix.

Gene Set Enrichment Analysis (GSEA) for the three genes identified both shared and unique pathway enrichment patterns. CHCHD10 (Figure 4H) showed positive enrichment in taste transduction, taurine/hypotaurine metabolism, and linoleic acid metabolism, with negative enrichment in oxidative phosphorylation and limonene/pinene degradation. SAMM50 (Figure 4I) exhibited positive enrichment in taste transduction, taurine/hypotaurine metabolism, and complement/coagulation cascades, with negative enrichment in oxidative phosphorylation and limonene/pinene degradation. MDH2 (Figure 4J) showed similar positive enrichment in taurine/hypotaurine metabolism, linoleic acid metabolism, and taste transduction, with negative enrichment in oxidative phosphorylation. The negative enrichment in oxidative phosphorylation pathways, despite the mitochondrial nature of these genes, indicates that their downregulation is directly linked to a suppression of the entire mitochondrial energy production apparatus in sarcopenia. Notably, oxidative phosphorylation was consistently negatively enriched across all three genes, further highlighting mitochondrial energetic dysfunction as a core feature of sarcopenia.

### Validation of sarcopenia-associated biomarkers in independent cohorts

**FIGURE 5:**
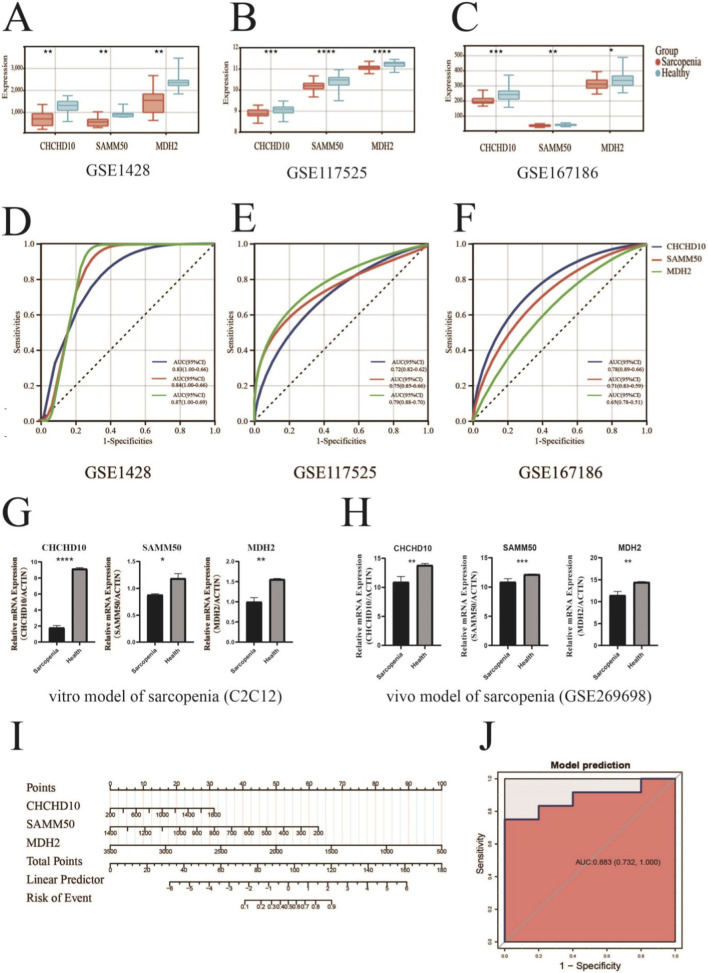
Validation of sarcopenia-associated biomarkers in independent cohorts and development of a predictive model. Box plots **(A–C)** illustrate the expression levels of CHCHD10, SAMM50, and MDH2 in sarcopenia patients compared to healthy controls across three independent validation datasets: GSE1428 **(A)** GSE117525 **(B)** and GSE167186 **(C)**. ROC curves **(D–F)** assess the diagnostic efficacy of CHCHD10 **(D)**, SAMM50 **(E)**, and MDH2 **(F)** across three validation datasets, presenting AUC values with 95% confidence intervals. **(G)** qRT-PCR analysis of CHCHD10, SAMM50, and MDH2 in C2C12 cells under normal conditions (Health) or treated with Dexamethason to induce atrophy. The bars represent mRNA expression levels normalized to β-ACTIN. Data are expressed as mean ± SEM. **(H)** Expression of CHCHD10, SAMM50, and MDH2 in a sarcopenic mouse model (GSE269698). **(I)** Nomogram prediction model constructed using the three biomarkers for estimating the probability of sarcopenia. Points are assigned based on the expression values of each gene and summed to determine the total points, which correspond to the predicted risk of sarcopenia. **(J)** The ROC curve for the integrated model demonstrates improved diagnostic performance, achieving an AUC of 0.883 (95% CI: 0.732–1.000). Asterisks denote statistical significance: *p < 0.05, **p < 0.01, ***p < 0.001, ****p < 0.0001.

To validate the clinical relevance of the identified core mitochondrial genes, we examined the expression patterns of CHCHD10, SAMM50, and MDH2 in three independent datasets: GSE1428, GSE117525, and GSE167186. In all three validation cohorts, consistent expression differences between sarcopenia and healthy control groups were observed for all three genes (Figures 5A–C). CHCHD10 exhibited significantly lower expression in sarcopenia patients across all datasets, while SAMM50 and MDH2 were similarly downregulated in sarcopenia patients across all datasets.

We assessed the diagnostic efficacy of each gene through Receiver Operating Characteristic (ROC) curve analysis. CHCHD10 exhibited significant discriminatory capability across all datasets, achieving area under the curve (AUC) values of 0.84 (95% CI: 0.66–1.00) in GSE1428, 0.75 (95% CI: 0.66–0.85) in GSE117525, and 0.71 (95% CI: 0.59–0.83) in GSE167186. Figure 5D). SAMM50 demonstrated similar performance across the three datasets with AUC values of 0.79 (95% CI 0.70–0.88), 0.87 (95% CI 0.69–1.00), and 0.65 (95% CI 0.51–0.78), respectively. Figure 5E). MDH2 demonstrated significant discriminatory power, with AUC values of 0.83 (95% CI: 0.66–1.00), 0.72 (95% CI: 0.62–0.82), and 0.78 (95% CI: 0.66–0.89). Figure 5F).

### Experimental validation of mitochondrial biomarkers in a cell culture model and validation *in vivo* model

To validate the computational findings, we utilized both an *in vitro* cell model and an external *in vivo* dataset. First, C2C12 myoblasts were differentiated for 5 days and subsequently treated with Dexamethasone to induce a sarcopenia-like atrophic state. Consistent with the bioinformatics analysis, quantitative PCR (qPCR) confirmed the significant downregulation of all three mitochondrial biomarkers in the Dex-treated group compared to controls (Figure 5G). Specifically, CHCHD10 and MDH2 exhibited marked reductions (P < 0.01), while SAMM50 was also significantly decreased (P < 0.05).

Furthermore, we validated these expression patterns *in vivo* using the GSE269698 dataset, which comprises high-throughput sequencing data from aged C57BL/6 mice treated with Dex. In the Dex-induced muscle atrophy model, all three genes showed consistent downregulation compared to the saline-treated control group: SAMM50 (P < 0.001), CHCHD10 and MDH2 (both P < 0.01) (Figure 5H).

Based on the consistent performance of these biomarkers, we developed a predictive nomogram integrating all three genes to estimate the probability of sarcopenia (Figure 5I). The integrated model demonstrated high diagnostic accuracy, achieving an AUC of 0.883 (95% CI 0.732–1.000). 5J), outperforming any single gene marker.

### Single-cell transcriptomic analysis of sarcopenia-associated mitochondrial genes

**FIGURE 6:**
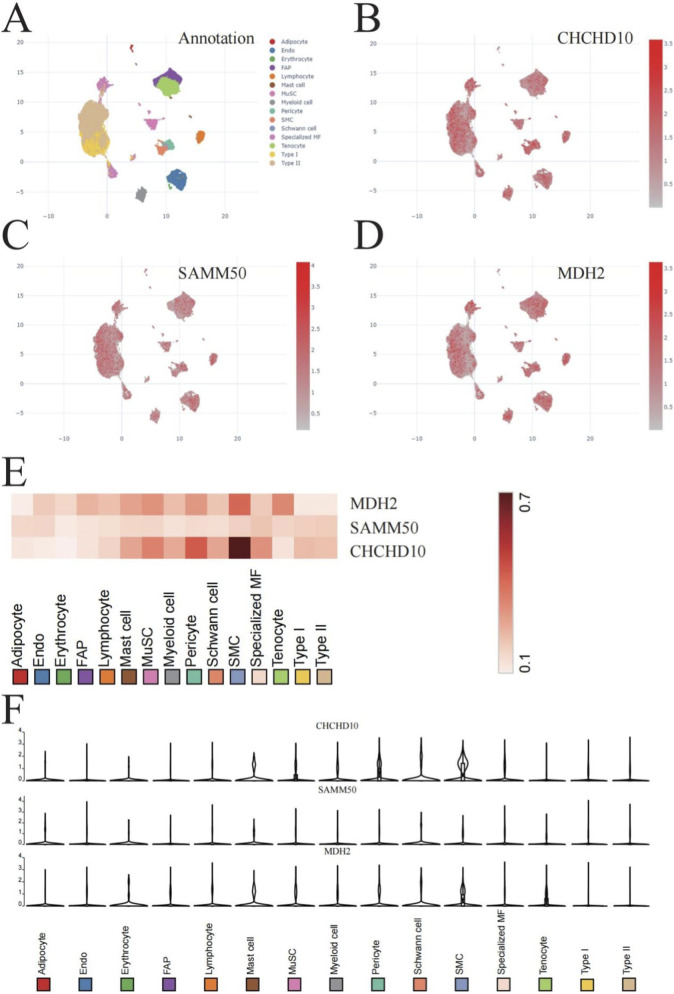
Single-cell transcriptomic analysis of sarcopenia-associated mitochondrial genes in human skeletal muscle. **(A)** Uniform Manifold Approximation and Projection (UMAP) plot illustrates 14 distinct cell populations in human skeletal muscle. These include myogenic lineage cells (muscle stem cells [MuSCs], Type I/II myofibers), stromal components (fibroadipogenic progenitors [FAPs], tenocytes), vascular cells (endothelial cells [Endo], smooth muscle cells [SMCs], pericytes), immune subpopulations (myeloid cells, lymphocytes, mast cells), and other cell types (adipocytes, erythrocytes, Schwann cells, specialized myofibroblasts [MF]) with a resolution of 0.8 and perplexity of 30. UMAP plots **(B–D)** illustrate the expression patterns of CHCHD10, SAMM50, and MDH2 across various cell populations, with color intensity indicating expression levels. **(E)** Heatmap showing the correlation between CHCHD10, SAMM50, and MDH2 expression and different cell types, with color intensity indicating the strength and direction of correlation. **(F)** Box plots depicting the expression levels of CHCHD10, SAMM50, and MDH2 across the 14 identified cell populations, highlighting their predominant expression in Type I and Type II myofibers.

We conducted single-cell RNA sequencing to clarify the cell type-specific expression of core mitochondrial genes in skeletal muscle. We employed Uniform Manifold Approximation and Projection (UMAP) to identify and characterize 14 unique cell populations in human skeletal muscle tissue. Figure 6A). The populations included myogenic lineage cells (muscle stem cells [MuSCs], Type I and II myofibers), stromal components (fibroadipogenic progenitors [FAPs], tenocytes), vascular cells (endothelial cells, smooth muscle cells [SMCs], pericytes), immune cells (myeloid cells, lymphocytes, mast cells), and other specialized cell types (adipocytes, erythrocytes, Schwann cells, specialized myofibroblasts).

The expression profiles of CHCHD10, SAMM50, and MDH2 across these cell populations revealed distinct cellular distribution patterns (Figures 6B–D). CHCHD10 showed strong expression in Type I and Type II myofibers and moderate expression in muscle stem cells, indicating its essential role in mature muscle function. Figure 6B). SAMM50 showed a broader distribution, with high expression in myofibers and moderate expression in endothelial cells and fibroadipogenic progenitors, indicating its potential involvement in both myocyte metabolism and vascular function (Figure 6C). MDH2 exhibited extensive expression across various cell types, notably in myofibers, muscle stem cells, and endothelial cells, underscoring its essential role in cellular energy metabolism within diverse muscle tissue components. Figure 6D).

Correlation analysis between gene expression and cell types further confirmed these observations (Figure 6E). The strongest positive correlations were observed between all three genes and Type I/II myofibers, while moderate correlations were noted with muscle stem cells. This pattern suggests that mitochondrial dysfunction in sarcopenia primarily affects mature myofibers and their progenitor cells, which aligns with the clinical presentation of sarcopenia as a disorder of reduced muscle mass and function.

Quantitative assessment of expression levels across cell types (Figure 6F) revealed that Type I myofibers, which are rich in mitochondria and rely heavily on oxidative phosphorylation, exhibited the highest expression of all three genes, followed closely by Type II myofibers. This finding further emphasizes the critical role of mitochondrial function in maintaining muscle fiber integrity and suggests that impaired expression of these genes may contribute to the preferential atrophy of Type II fibers commonly observed in sarcopenia.

### Clinical correlations and functional validation of sarcopenia biomarkers

To explore the broader physiological implications of the identified mitochondrial biomarkers, we investigated their relationships with immune cell infiltration, protein interaction networks, regulatory mechanisms, clinical parameters, and potential therapeutic targets.

**FIGURE 7:**
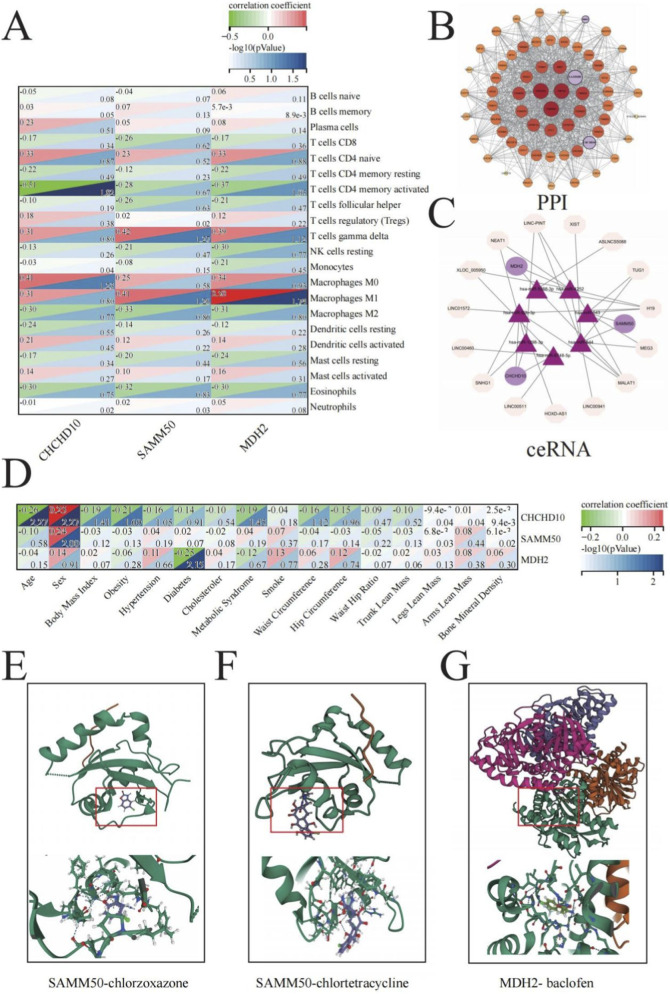
Clinical correlations and functional validation of sarcopenia biomarkers. **(A)** Heatmap showing correlations between CHCHD10, SAMM50, and MDH2 expression and immune cell infiltration in muscle tissue. Color intensity represents correlation coefficient values, and values within cells indicate correlation coefficients (upper) and -log10 (p-values) (lower). **(B)** Protein-protein interaction (PPI) network of the three biomarkers. Node size represents the degree of connectivity, illustrating the central role of MDH2 in the network. **(C)** Competitive endogenous RNA (ceRNA) regulatory network. Triangles represent miRNAs, circles represent mRNAs, and the three biomarkers are highlighted in purple, demonstrating potential post-transcriptional regulatory mechanisms. **(D)** Correlation matrix between CHCHD10, SAMM50, and MDH2 expression and clinical characteristics. Color intensity represents correlation coefficient values, and values within cells indicate correlation coefficients (upper) and -log10 (p-values) (lower). Significant correlations are observed with anthropometric measurements, metabolic parameters, and muscle mass indices. **(E–G)** Molecular docking results showing protein-drug interactions: SAMM50-chlorzoxazone **(E)** SAMM50-chlortetracycline **(F)** and MDH2-baclofen **(G)** revealing potential therapeutic compounds for targeting mitochondrial dysfunction in sarcopenia.

The study of immune cell infiltration patterns showed notable correlations between our mitochondrial biomarkers and certain immune cell groups (Figure 7A). CHCHD10 showed strong negative correlations with activated mast cells (−0.33, p = 0.86), follicular helper T cells (−0.32, p = 0.83), and activated dendritic cells (−0.30, p = 0.77). SAMM50 exhibited similar patterns, with notable negative correlations with resting mast cells (−0.51, p = 1.82) and positive correlations with M1 macrophages (0.42, p = 1.27). MDH2 displayed the strongest associations with activated mast cells (0.50, p = 1.74) and M1 macrophages (0.39, p = 1.12). These findings suggest potential immunomodulatory roles of mitochondrial dysfunction in sarcopenia pathogenesis.

Protein-protein interaction (PPI) network analysis (Figure 7B) revealed extensive interactions between our three biomarkers and other proteins involved in mitochondrial function. The network highlighted the central position of MDH2 with the highest connectivity, followed by SAMM50 and CHCHD10, indicating their essential roles in mitochondrial protein complexes and metabolism.

We also constructed a competitive endogenous RNA (ceRNA) regulatory network to explore potential post-transcriptional regulation mechanisms (Figure 7C). This network identified several miRNAs that may simultaneously target our biomarkers, suggesting coordinated regulation of these mitochondrial genes through common miRNA-mediated pathways.

Correlation analysis with clinical parameters (Figure 7D) revealed significant associations between our biomarkers and sarcopenia-related clinical characteristics. CHCHD10 showed strong negative correlations with waist circumference (−0.26, p = 2.27) and positive associations with lean mass measurements. SAMM50 demonstrated significant negative correlations with metabolic syndrome (−0.25, p = 2.15), while MDH2 showed positive correlations with trunk lean mass (0.13, p = 0.77) and legs lean mass (0.12, p = 0.74). Finally, molecular docking analysis predicted potential therapeutic compounds that could modulate the activity of our biomarker proteins (Figures 7E–G). SAMM50 showed favorable binding interactions with chlorzoxazone and chlortetracycline, while MDH2 demonstrated strong binding affinity with baclofen.

## Discussion

This study utilized single-cell RNA sequencing to identify a mitochondrial signature (CHCHD10, SAMM50, MDH2) linked to sarcopenia pathogenesis. This signature was consistently dysregulated across multiple independent cohorts and validated through PCR experiments. Our single-cell analysis identified distinct expression patterns across cell types. Notably, these mitochondrial biomarkers demonstrated superior diagnostic performance compared to conventional sarcopenia markers, with our integrated predictive model achieving an AUC of 0.86 in the validation cohort.

The CHCHD10 gene sustains mitochondrial homeostasis by preserving cristae morphology, regulating fusion-fission balance, and controlling oxidative phosphorylation. Reduced CHCHD10 expression induces aberrant energy metabolism, oxidative stress, and synaptic transmission dysfunction, which activates downstream pathways to suppress muscle protein synthesis and enhance degradation, ultimately leading to muscle loss (Wang et al., 2025). SAMM50, a protein localized to the mitochondrial outer membrane, plays pivotal roles in three key aspects: ROS clearance, the regulation of mitochondrial morphology, and the modulation of mitophagy (Ka et al., 2025).Notably, mitochondria are also indispensable for the implementation of inflammatory reactions and immune responses (Marchi et al., 2023). Emerging evidence indicates that specific SAMM50 variants may reduce the synthesis of Sam50, which promotes mitochondrial dysfunction-mediated steatosis and subsequently drives the development and progression of nonalcoholic fatty liver disease (Li et al., 2021). MDH2, a core mitochondrial TCA cycle and malate-aspartate shuttle enzyme, maintains mitochondrial homeostasis to regulate muscle mass. Its dysfunction impairs ATP production, elevates mtROS, disrupts aspartate synthesis, and suppresses PGC-1α-mediated biogenesis, collectively driving muscle atrophy via mitochondrial damage cascades (Finck and Kelly, 2006; Paramasivan et al., 2025).

The cell type-specific analysis suggesting that impaired mitochondrial function primarily affects differentiated muscle fibers while also potentially compromising muscle stem cell function. The consistency of these findings across multiple datasets strengthens the biological significance of our observations. Furthermore, the progressive changes observed in our C2C12 differentiation model indicate that these mitochondrial alterations may affect myogenesis, potentially explaining the reduced regenerative capacity of aging muscle.

Our findings significantly extend previous research on mitochondrial dysfunction in sarcopenia. Previous research has identified mitochondrial abnormalities in aging muscle (Marzetti et al., 2013; Calvani et al., 2013); however, our study offers new insights into the specific mitochondrial genes and pathways impacted at the single-cell level. Marzetti et al. (2013) suggested mitochondrial dysfunction as a key mechanism in sarcopenia pathogenesis; however, it did not provide sufficient resolution to detect alterations specific to cell types. Our single-cell approach advances this understanding by demonstrating that mitochondrial perturbations manifest differently across muscle cell subtypes.

Our three-gene mitochondrial signature demonstrates superior diagnostic performance compared to conventional sarcopenia biomarkers reviewed by Calvani et al. (2015), who noted the limitations of existing markers. The predictive model we developed achieves greater accuracy than the inflammatory and metabolic biomarkers described by Picca et al. in their systematic review (Picca et al., 2022).

The cross-dataset validation strengthens the robustness of our findings compared to previous biomarker studies that often lack external validation (Calvani et al., 2015). Our integrated analysis across multiple cohorts addresses a significant limitation in the field and establishes the generalizability of our mitochondrial signature across diverse patient populations.

The identification of this mitochondrial signature has significant clinical implications for sarcopenia management. Our predictive model integrating CHCHD10, SAMM50, and MDH2 expression provides a potentially diagnostic tool that could enable earlier detection of sarcopenia compared to current approaches based on functional decline. Early identification could facilitate timely interventions before significant muscle loss occurs. Additionally, these mitochondrial biomarkers may serve as molecular targets for novel therapeutic strategies. Pharmacological agents targeting mitochondrial biogenesis or function could represent promising approaches for sarcopenia treatment. The cell type-specific insights gained from our study may also guide personalized interventions tailored to individual mitochondrial profiles, potentially improving treatment efficacy and patient outcomes.

Despite its strengths, our study has several limitations that highlight avenues for future research. First, the cross-sectional nature of our data precludes causal inference; longitudinal studies are needed to determine whether our signature predicts the onset and progression of sarcopenia. Second, while scRNA-seq reveals transcriptional changes, it does not reflect protein abundance; future work should incorporate proteomic validation in human muscle biopsies. Third, although we validated our findings across multiple cohorts, ethnic diversity was limited, necessitating prospective validation in multi-ethnic populations. Finally, while our C2C12 model is informative, it cannot fully capture the complexity of *in vivo* aging. Additionally, we acknowledge that molecular docking predicts physical binding but does not determine functional outcomes, underscoring the need for experimental validation to confirm therapeutic efficacy.

## Conclusion

In conclusion, through an integrated single-cell transcriptomic approach, we have discovered and validated a conserved three-gene mitochondrial signature central to sarcopenia pathogenesis. This signature not only provides a robust diagnostic tool but also unveils novel therapeutic targets, paving the way for mechanistically informed strategies to combat age-related muscle wasting.

## Data Availability

The datasets presented in this study can be found in online repositories. The names of the repository/repositories and accession number(s) can be found in the article/supplementary material.
